# Public health round-up

**DOI:** 10.2471/BLT.15.010515

**Published:** 2015-05-01

**Cheap but lethal: illicit tobacco**

This year’s No Tobacco day on 31 May highlights the way the tobacco industry and criminal groups profit from the illegal trade in tobacco, leaving the public to pay the health and security costs.

http://www.who.int/campaigns/no-tobacco-day/2015/event

**Figure:**
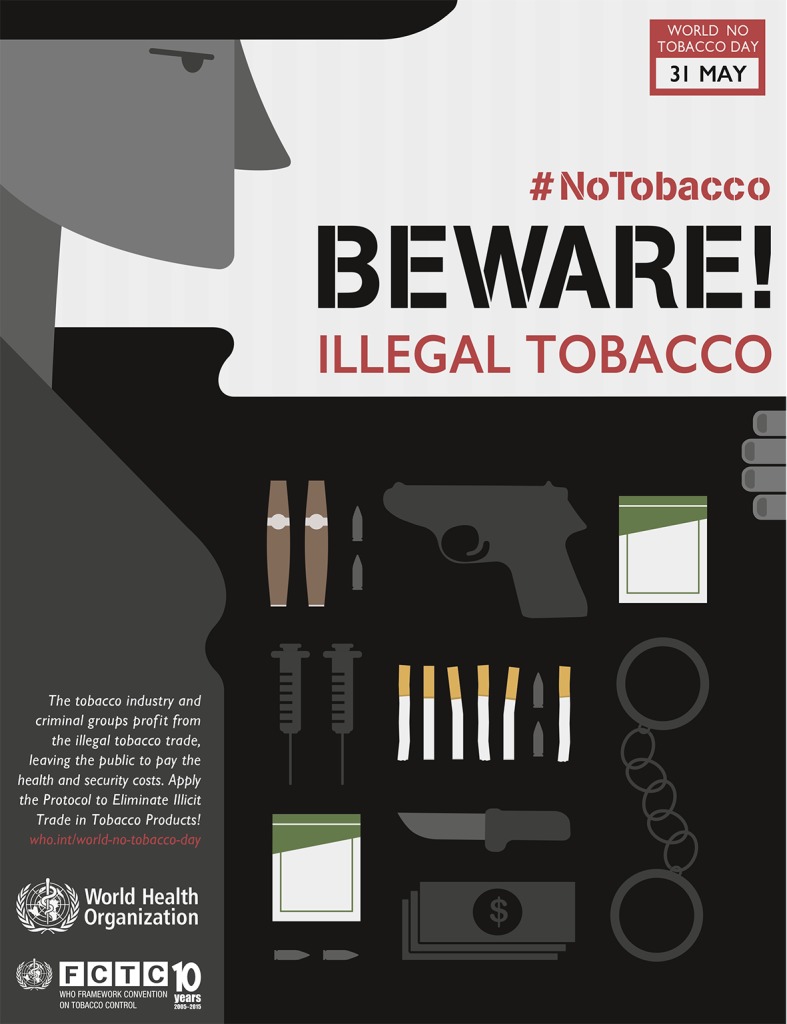
WHO

## Guineans test Ebola vaccine

Ring vaccination of the last few participants in the first trial testing the efficacy of a new Ebola vaccine in the western African epidemic is due to be completed next month.

Ring vaccination involves identifying people who have been recently infected with the virus and vaccinating those who have been in contact with them, thus creating a ring of immunity to stop further spread of the virus in the community.

The trial started on 7 March in areas of Basse Guinée, a region with one of the highest number of cases in the country and ring vaccination started there on 23 March. The aim is to vaccinate about 10 000 adults in 190 rings over a period of 8 to 10 weeks.

Researchers are testing the VSV-EBOV vaccine, which was developed by the Public Health Agency of Canada and licensed to NewLink Genetics in the United States of America (USA). Last year, Newlink announced its collaboration with Merck, which is taking responsibility for the future development of the vaccine.

The trial is being organized by the Guinean government, in collaboration with the World Health Organization (WHO) and Médecins Sans Frontières.

Once vaccinated, participants in the Basse Guinée region will be followed for three months and the results could be available as early as July or August.

“This landmark operation gives hope to all of us in Guinea and in the world that we might soon have an effective public health tool against Ebola, should the vaccine prove to be safe and effective,” said Dr Jean-Marie Dangou, the WHO Representative in Guinea.

The ring vaccination trial design was developed by an international group of experts from Canada, France, Guinea, Norway, Switzerland, the United Kingdom of Great Britain and Northern Ireland and the United States of America in collaboration with WHO. They include Professor Donald A Henderson, who led WHO’s global smallpox eradication programme from 1966 to 1977.

http://www.who.int/medicines/ebola-treatment/q_a_vaccine_trial_guinea

## Foreign medical teams

WHO has started to build a global roster of foreign medical teams ready to deploy in emergencies.

Under the new mechanism, WHO sets minimum standards for the international health workers that are listed in the Global Foreign Medical Teams Registry, requiring that health personnel and medical teams provide a clear description of the services and skills that they can offer.

Their credentials are verified and they are classified according to their skills, so that WHO can search for teams with the required skill set and they can be deployed to health emergencies at short notice.

“WHO’s work to improve the global response to emergencies has benefits for all countries,” said Dr Ian Norton, who leads the work on foreign medical teams at WHO.

“Thanks to the mechanism we have developed, the international response to the cyclone in Vanuatu has been very fast and efficient,” he said. 

The first medical team arrived from Australia to support the local health workers just 2 days after the cyclone hit. Since then, 20 teams (including more than 50 doctors, 40 nurses, 24 paramedics and 12 midwives) have provided assistance. New teams continue to arrive to fill positions as other teams return to their home countries.

The new deployment and coordination mechanism can also cover teams providing clinical care in emergencies such as tsunamis, earthquakes and flooding, as well as large outbreaks, such as the Ebola virus disease in western Africa, which require a surge in clinical-care capacity. The mechanism is part of WHO’s plans for a Global Emergency Workforce.

http://www.who.int/hac/techguidance/preparedness/foreign_medical_teams

**Cover photo**

Students walk to Gyezmo Primary School in Nigeria’s Bauchi State. Their school is among those that have benefited from the Girls’ Education Project that is led by the Nigerian Government with support from the United Kingdom’s Department for International Development and the United Nations Children’s Fund (UNICEF).

**Figure:**
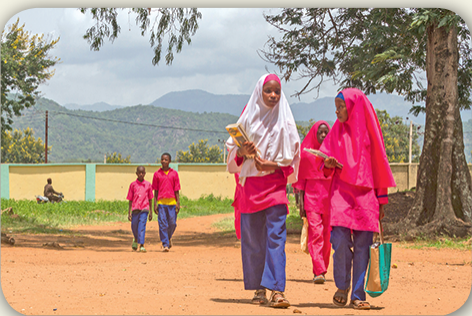
© UNICEFNYHQ2014Esiebo

## All trials, all results

WHO called on the scientific community to disclose the results of all clinical trials for medical products, regardless of the result. 

The call, in a public statement issued last month, applies to current medical research as well as unreported trials conducted in the past because the results may still be relevant.

The aim is to ensure that all evidence related to the safety and efficacy of vaccines, drugs and medical devices for use by populations is made publicly available. Thus, health policies can be based upon the best available evidence. 

“We want to promote the sharing of scientific knowledge to advance public health,” said Dr Marie-Paule Kieny, WHO Assistant Director-General for Health Systems and Innovation.

“Failure to publicly disclose trial results leads to misinformation and skewed priorities for research and development and for public health interventions,” said Kieny. “This failure also creates indirect costs for public and private entities, including the patients themselves, who may end up paying for suboptimal or harmful treatments.”

In 2005, WHO called on the scientific community to ensure that all clinical trials are registered on a WHO primary clinical trial registry.

It also established the International Clinical Trials Registry platform that regularly imports trial records from primary trial registries and data providers, such as Clinicaltrials.gov.

Despite the 2005 initiative, researchers still do not always publish their results. For example, a study of large clinical trials – with more than 500 participants – registered on clinicaltrials.gov and completed by 2009, showed that 23% had not reported their results.

http://www.who.int/ictrp/results/reporting

## Health crisis in Yemen

WHO has been working with the health ministry and its partners to respond to the urgent health needs of the population since the conflict escalated in March.

Hundreds of people have died and thousands have been injured. Medical supplies and medicines have become increasingly scarce across the country and health facilities in the affected areas – some working at reduced capacity – have been overwhelmed with casualties.

In response, WHO has provided eight interagency health kits for 240 000 people from its warehouses in Sana’a, Aden and Hodeidah, as well as trauma kits for 400 operations, 11 000 blood bags, intravenous fluids, analgesics, oxygen supplies and dressing materials to 18 hospitals throughout the country.

WHO is also in the process of locally procuring an additional 10 trauma kits for 1000 operations. To maintain the cold chain for vaccines in health facilities in spite of power cuts in Aden, WHO was working with the health ministry to procure generator sets locally.

WHO and the health ministry sent ambulances to parts of the country where they are needed most, while WHO covered the operational costs of the ambulances and installing GPS tracking devices in them to prevent misuse. Additional ambulances are also needed.

“We have been able to respond to most reported shortages for the time being, but the needs are huge and the sooner we are allowed to send additional supplies into the country without restrictions in access, the more lives we can save,” said Dr Ahmed Shadoul, the WHO Representative to Yemen.

As health needs grow, WHO has also pre-positioned additional interagency health kits and trauma kits in its humanitarian hub in Dubai for transporting to Yemen when the security situation permits. 

http://www.emro.who.int/yem/yemen-news/who-responds-to-urgent-health-needs-in-yemen.html

## Foodborne diseases

There were an estimated 582 million cases of 22 different foodborne enteric diseases and 351 000 associated deaths in 2010, according to initial findings from WHO’s Foodborne Disease Burden Epidemiology Reference Group (FERG).

These figures are among the first findings from an ongoing estimation of the global burden of foodborne disease caused by a number of viruses, bacteria, parasites, chemicals and toxins. 

These first figures relate to enteric infections – those affecting the intestines – caused by viruses, bacteria and protozoa that enter the body when people eat contaminated food. The new figures were released on 7 April, World Health Day, which was devoted to food safety this year.

Further estimates show that the foodborne enteric disease agents responsible for the most deaths are: Salmonella Typhi (52 000 deaths), enteropathogenic *Escherichia coli* (37 000) and norovirus (35 000).

The WHO African Region recorded the highest incidence of enteric foodborne disease, followed by the WHO South-East Asia Region.

More than 40% of those suffering from enteric diseases caused by contaminated food are children aged less than 5 years of age.

In 2006, WHO launched an initiative to estimate the global burden of foodborne diseases to provide its Member States with data and tools that these governments can use to develop evidence-based policies on food safety.

The FERG was established in 2008 and has produced several sets of global estimates on foodborne disease, including on diarrhoea in children, human brucellosis and listeriosis.

The full results of the study of foodborne enteric diseases are due to be released in September. 

http://www.who.int/foodsafety/areas_work/foodborne-diseases/ferg

**Looking ahead**

**18–26 May – World Health Assembly**. http://www.who.int/mediacentre/events/governance/wha
**31 May – World No Tobacco Day 2015: stop illicit trade of tobacco products**. http://www.who.int/campaigns/no-tobacco-day/2015/event
**9–11 June – High-level International Conference on the Implementation of the Water for Life Decade**. Dushanbe, Tajikistan. http://waterforlifeconf2015.org/eng/obosnovanie
**14 June – World Blood Donor Day**. http://www.who.int/campaigns/world-blood-donor-day/2015/event
**13–16 July – 3rd International Conference on Financing for Development**. Addis Ababa, Ethiopia. http://www.un.org/esa/ffd/overview/third-conference-ffd.html
**28 July – World Hepatitis Day**.
**25–27 September – United Nations Summit to adopt the post-2015 development agenda**. New York, USA.

